# The Role of *Chrysoperla carnea* (Steph.) (Neuroptera: Chrysopidae) as a Potential Dispersive Agent of Noctuid Baculoviruses

**DOI:** 10.3390/insects11110760

**Published:** 2020-11-05

**Authors:** Oscar Giovanni Gutiérrez-Cárdenas, Ángeles Adán, Inés Beperet, Pilar Medina, Primitivo Caballero, Agustín Garzón

**Affiliations:** 1 Crop Protection Unit, School of Agricultural, Food and Biosystems Engineering, Technical University of Madrid, Puerta de Hierro, 2, 28040 Madrid, Spain or og.gutierrez@alumnos.upm.es (O.G.G.-C.); angeles.adan@upm.es (Á.A.); pilar.medina@upm.es (P.M.); 2 Research & Development Department, Bioinsectis SL. Pol. Ind. Mocholi Plaza Cein 5, Nave A14, 31110 Noain, Navarra, Spain; ines.beperet@unavarra.es; 3 Institute for Multidisciplinary Research in Applied Biology, Public University of Navarre, 31006 Pamplona, Navarra, Spain; pcm92@unavarra.es

**Keywords:** AcMNPV, excretion products, horizontal transmission, occlusion bodies, SeMNPV

## Abstract

**Simple Summary:**

Baculoviruses (BV) infect several lepidopteran pests of economic importance, such as the beet armyworm *Spodoptera exigua*. The joint use of microbiological and macrobiological strategies may improve the efficacy of control. Laboratory bioassays were developed to evaluate the interactions between two BVs: the multiple nucleopolyhedroviruses of *S. exigua* (SeMNPV) and *Autographa californica* (AcMNPV), and the predator *Chrysoperla carnea*. The excretion products of the predator’s larvae (drops) and adults (meconia) were microscopically examined after the ingestion of BV-infected *S. exigua* larvae. For both types of excreta and BVs, viral occlusion bodies (OBs) (resistance forms) were observed. These OBs were infective to healthy *S. exigua* larvae when applied in water suspension and in direct deposition. The virulence of meconia was higher in suspensions (higher viral load), while larval drops were more virulent in direct application due to their liquid nature and their easiness of consumption. The fitness of *C. carnea* was slightly affected by the consumption of both BV-infected prey. No preference was shown between healthy and BV-infected *S. exigua*, and both were preferred vs. the aphid *Macrosiphum euphorbiae*. Our findings present *C. carnea*, and particularly its larvae, as a promissory candidate for BV dispersion in the field.

**Abstract:**

Baculoviruses (BV) are highly effective against lepidopteran pests of economic importance such as *Spodoptera exigua*. The combined use of entomopathogens and macrobiological control agents requires the study of their relationships. Laboratory bioassays were developed to evaluate the interactions between the multiple nucleopolyhedroviruses of *S. exigua* (SeMNPV) and *Autographa californica* (AcMNPV), and the predator *Chrysoperla carnea*. The microscopic examination of predator’s excreta (larval drops and meconia) after the ingestion of BV-infected *S. exigua* revealed the presence of viral occlusion bodies (OBs). The reinfection of *S. exigua* larvae with BVs-contaminated excreta by using OBs water suspensions or by direct application both yielded high mortality values but different speed-of-kill results. Meconia killed before in suspensions due to their higher viral load and larval excretion drops did so in direct application due to their liquid nature and their easiness of consumption. The prey-mediated ingestion of SeMNPV and AcMNPV triggered slight effects in *C. carnea*, which were probably derived from the food nutritional quality. *Chrysoperla carnea* larvae did not discriminate between healthy and BV-infected *S. exigua*, while a preference was shown for *S. exigua* (healthy or infected) vs. *Macrosiphum euphorbiae*. Our findings present *C. carnea*, and particularly its larvae, as a promissory candidate for BV dispersion in the field.

## 1. Introduction

Amongst insect pathogens, the family Baculoviridae comprises a large number of pathogenic viruses. As components of ecosystems, baculoviruses (BV) are important agents in the demographic regulation of a few hundred insect species, including some of the most economically important lepidopteran pests [1,2,3]. Furthermore, BV-based insecticides are highly effective for the control of a growing number of lepidopteran crop pests. Its commercial development has steadily increased along the last decades with up to 50 BV-based commercial products [2].

In contrast to chemical pesticides, BV act as ecological entities that have the potential to infect, multiply, spread (both horizontally and vertically), and persist on plants and soil [4]. Amongst the dissemination strategies of BV, the horizontal transmission by the release of occlusion bodies (OBs) from infected corpses represents the main spreading path [1,5]. Predators, which are more mobile than herbivorous caterpillars, may also contribute to the dispersal of BV because they excrete viable viral OBs after feeding on infected prey [5,6,7]. Studies under semi-field and field conditions suggest that several insect groups with zoophagous habits promote a faster virus dissemination [5,8]. A complete understanding of the existing interactions among BV and predators is currently lacking in the scientific literature. In fact, shedding light on these issues has an evident practical application, because the release of predators with entomopathogenic OBs in their digestive tract would allow the combination of macro- and microbiological control in one single step, therefore reducing the crop inputs.

Larvae of the lacewing *Chrysoperla carnea* Stephens (Neuroptera: Chrysopidae) are considered important naturally occurring predators in many agricultural cropping systems. In addition, it is one of the most commonly available natural enemies in bio-factories [9]. Although larval feeding preferences are mainly homopterophilic [10], the diversity of its potential prey is wide, predating also lepidopteran eggs and caterpillars [11,12,13].

*Chrysoperla carnea* has two types of excretion products (liquid and solid) depending on the developmental stage of its biological cycle. The digestive tube of larvae is closed behind the midgut, and therefore, they only excrete drops containing the soluble remains of digestion that are drained off by the Malpighian tubules. On the other hand, the insoluble remnants of prey consumed during the larval period are stored until the emergence of adults and ejected once as solid debris: the meconium [9,10].

So far, the presence of OBs in the meconia of adult individuals of lacewing species has been assessed as well as their potential to reinfect noctuid larvae [14,15]. Nevertheless, their detection in the excretion products of lacewing larvae and their viability to cause pest reinfection has been scarcely studied [16], despite representing an alternative spreading path with even higher infective potential in field conditions compared to meconia. The active prey-searching behavior of lacewing larvae [10], together with the continuous deposition of excretion drops on crop surfaces, are factors involved in this expected potential [17]. The liquid nature of larval debris in comparison with a solid and dry adult meconium increases the possibilities of being ingested by the early instars of caterpillars.

This study examines the presence and activity of the OBs in the larval and adult excretion products of the predator *C. carnea* when used as prey for *Spodoptera exigua* Hübner (Lepidoptera: Noctuidae) larvae that are infected by two alphabaculoviruses (the multiple nucleopolyhedroviruses of *S. exigua* (SeMNPV) and *Autographa californica* (AcMNPV)). Despite being baculoviruses of the same phylogenetic group and sharing *S. exigua* as a common host, these alphabaculoviruses have different physical and biological properties that may affect in some manner the interaction with the predator [16,18,19]. In addition, other predator–pathogen interactions have been also evaluated: the predator’s preference between healthy and BV-infected prey and the effect of the consumption of BV-infected *S. exigua* larvae on the predator’s fitness.

## 2. Materials and Methods

### 2.1. Biological Material

The rearing of insects and the bioassays were developed in the Universidad Politécnica de Madrid (UPM), Spain. The climatic chambers conditions were controlled at 25 ± 2 °C with relative humidity (RH) at 65% ± 10%, and a photoperiod of 16:8 (light:dark) h.

#### 2.1.1. Viruses

Two baculovirus isolates provided by the Professor Primitivo Caballero from the Universidad Pública de Navarra (UPNA), Spain, were used. These isolates correspond to multiple nucleopolyhedrovirus of *Spodoptera exigua* (SeMNPV) and multiple nucleopolyhedrovirus of *Autographa californica* (AcMNPV). Both baculovirus isolates were produced in fourth-instar *S. exigua.* The virus suspensions were purified, and OBs concentrations were determined with the help of an improved hemocytometer (Hawksley Ltd., Lancing, UK) at UPNA before their use in the bioassays.

#### 2.1.2. Insects

A laboratory rearing of *S. exigua* was established with larvae provided by the UPNA. Larvae were fed ad libitum with the artificial diet described by [20] until pupation. In adult rearing cages, a solution of water + honey (30% *v*/*v*) was used as a food source, and filter paper was placed inside for oviposition purposes. For the trials, paper with <24-h-old eggs was used, and after hatching, larvae were left to develop until baculovirus inoculation at newly molted L_2_ instar. A laboratory colony of *C. carnea* was initiated with insects obtained from Agrobío^®^ (CHRYSOcontrol^®^, Almería, Spain), following the rearing procedure of [21]. Larvae were fed ad libitum with a diet based on a mixture of eggs of *Ephestia kuehniella* Zeller (Lepidoptera: Pyralidae) and *Artemia* spp. (1:5 *w*/*w*) provided by Koppert^®^ (Berkel en Rodenrijs, The Netherlands). For the trials, eggs (<24-h-old) were selected and after hatching, larvae were reared until L_3_ instar.

### 2.2. Presence of the OBs in Drop and Meconium

*Chrysoperla carnea* L_3_ (<24 h) were fed until pupation with larvae of *S. exigua* (L_2_, 3 days post inoculation (dpi)) from three sanitary conditions: (1) healthy larvae; (2) SeMNPV-infected larvae (LC_95_ = 1.9 × 10^5^ OBs·mL) and (3) AcMNPV-infected larvae (LC_95_ = 1.6 × 10^6^ OBs·mL). BV-infected larvae were inoculated with the droplet feeding method [22]. *S. exigua* larvae were previously starved for 24 h, and to guarantee the ingestion of virus solution, sucrose (15% *w*/*v*) was added to the solution as a food attractant, and food-grade blue color was added as an indicator (ProGel^®^, Preston, UK). Each sanitary condition of *S. exigua* was considered as a treatment, and each tested predator larva was considered a replicate (*n* = 56 per treatment). Each individual predator larva was offered a sufficient number (*n* = 10, based on previous experience) of *S. exigua* larvae daily and allowed to feed on them ad libitum. Non-consumed larvae were removed and replaced by a new batch of 10 individuals on a daily basis. This procedure was carried using as arenas blisters with 28 alveoli each (Arapack^®^, Zaragoza, Spain). The droplets excreted by the L_3_ larvae were obtained as dry residues from the blister lid once the larva had pupated with the help of a small scissor to cut the stained area. In addition, the meconia were also recovered with soft clamps after adult emergence. The pieces of cut lid and the meconia were individually introduced in Eppendorf^®^ tubes with 100 µL of sterile distilled water, and suspensions were shaken for 10 s with a vortex (Genie2^®^ Scientific Industries, New York, NY, USA). For both SeMNPV and AcMNPV, a total of 12 samples of excreted drops and meconia, respectively, were analyzed to quantify the concentration of OB supensions with an optical microscope (400× magnification lens, Leica, Microsystems GmbH, Wetzlar, Germany) using a Neubauer chamber (Reichert Scientific Instruments, Buffalo, NY, USA).

### 2.3. Infection of Healthy L_2_ S. exigua with Suspensions of OBs Obtained from C. carnea Excretion Products

Starting from the OBs suspensions obtained, an artificial suspension was prepared by mixing 20 µL of each sample. The final volume (240 µL) was mixed up with 60 µL of sterile distilled water with sucrose (15% *w*/*v*) and ProGel^®^ food-grade blue dye, obtaining a final volume of 300 µL (SeMNPV: 8.47 × 10^5^ OBs·mL^−1^ in drop, 4.09 × 10^6^ OBs·mL^−1^ in meconium; AcMNPV: 6.87 × 10^5^ OBs·mL^−1^ in drop, 4.12 × 10^6^ OBs·mL^−1^ in meconium). This suspension was used to perform a quantitative bioassay by using newly molted (<24 h) L_2_ *S. exigua* larvae following the droplet-feeding method described above. Four replicates of seven individuals were performed for a negative control (healthy *S. exigua* larvae) and for each route of excretion of OBs (drop and meconium) and baculovirus (SeMNPV and AcMNPV), daily assessing the BV-associated mortality until all the larvae died or pupated.

### 2.4. Infection of Healthy L_2_ S. exigua via Direct Deposition of C. carnea Excretion Products

This assay was performed to test the infectivity of the excretion products without being previously suspended in water, because in this way, this procedure is a more realistic approach at the *S. exigua* larvae infection under field conditions. For this purpose, L_3_ *C. carnea* larvae were fed for 2 days ad libitum with infected *S. exigua* larvae following the method described above and then individually left for 30 min on alveoli provided with *S. exigua* artificial diet to allow surface contamination with droplets excreted by predator larvae. This procedure was repeated with a second batch of *C. carnea* to ensure the presence of drops on the diet (checked by the observation of reddish stained areas). The same *C. carnea* larvae were retired and fed again with infected *S. exigua* larvae until pupation. When the adults emerged, the meconia were collected. Then, each meconium was placed on a new alveoli provided with an *S. exigua*-artificial diet. Once the *C. carnea* excretion products (drops and meconia) were respectively placed in the alveoli, one newly molted (<24 h) L_2_ *S. exigua* larva was introduced per alveolus, performing 4 replicates of 7 larvae for SeMNPV and AcMNPV respectively; a negative control was also performed. Larval BV-associated mortality was daily recorded until death or pupation of the survivors.

### 2.5. Chrysoperla carnea Fitness Evaluation after Prey-Mediated Ingestion of SeMNPV and AcMNPV

In addition to the recovering of the excretion products, the effects of consuming infected *S. exigua* larvae in predator’s fitness were assessed. The following parameters were recorded: daily consumption rate, L_3_ instar developmental time, percentage of pupation, pupal developmental time, pupal weight, percentage of adult emergence, and adult weight. After adult emergence, a number of 12 replicates of one couple (1♀ + 1♂) were formed per treatment and placed in plastic cages (19.5 × 16.5 × 8 cm), providing food and water to evaluate the reproductive parameters according to [23]. The preoviposition period was determined for each female. A piece of cotton gauze was placed in the upper part of the plastic cages, where the eggs laid by the *C. carnea* female were collected to measure fecundity. The reproduction assessment started one day after the first oviposition was observed and lasted for 14 days to cover a representative interval. Along this period, the daily oviposition of each replicate of 6 distributed samples were incubated (25 ± 2 °C, 65% ± 10% RH, photoperiod 16:8 h (L:D)) until hatching in separate plastic cages (9 cm ø, 2 cm height) to evaluate the fertility [24].

### 2.6. Chrysoperla carnea Choice Tests

Choice tests were conducted to address whether *C. carnea* larvae preferred BV-infected or healthy prey. *Spodoptera exigua* larvae (L_2_, <24 h) were inoculated with a virus suspension corresponding to the LC_95_ of SeMNPV or AcMNPV and 3 dpi were offered to lacewing L_3_ larvae. Healthy *S. exigua* larvae (L_2_, <72 h) or apterous adults of *Macrosiphum euphorbiae* Thomas (Hemiptera: Aphididae) were offered as alternative prey. *C. carnea* larvae (L_3_) were starved for 24 h prior to the experiments to increase their prey searching. The experimental arena consisted of a glass Petri dish (100 mm ø) with infected and healthy prey placed at opposite sides with freedom of movements within the arena. In the central point of the Petri dish a *C. carnea* larva was released and observed for 30 min to determine the type of prey selected and consumed (first attack); the time spent until the first attack occurred was also recorded (searching time). If no response was obtained after that period, the replicate was discarded. The bioassay consisted of 40–42 replicates with response for SeMNPV and AcMNPV, respectively. In addition, the preference of *C. carnea* between both healthy prey (uninfected L_2_ *S. exigua* or *M. euphorbiae* apterous adults) was also recorded (46 replicates).

### 2.7. Statistical Analysis

Data of OBs quantification were compared with a two-way analysis of variance. In the virus transmission assays, to determine the effects of the excretion products (drops and meconia) and BV (SeMNPV and AcMNPV), mortality data were subjected to Kaplan–Meier survival analysis. Categorical data were analyzed with a Chi-square (*X*^2^) test: the results of the choice tests with a Chi Square Goodness of Fit (One Sample Test) assuming a null hypothesis of no preference among categories; data without replicates (survival until pupation and emergence, and sexual ratio) with a 2 × 2 contingency Chi-square. Differences in the response variables of parameters considered in the fitness were assessed using one-way analysis of variance. The analysis was performed with SPSS Statistics Software Package for Windows version 24.0.0.0 [25].

## 3. Results

### 3.1. Presence of OBs in C. carnea Excretion Products

The presence of OBs in both excretion products of *C. carnea*, when fed on infected *S. exigua* larvae, was confirmed by the direct observation of samples under a light microscope. The number of OBs·mL^−1^ determined significantly differed depending on the excretion path (F_1,44_ = 146.78; *p* < 0.001) but was not affected by the virus causing the infection (F_1,44_ = 0.451; *p* = 0.505). The interaction was not significant (F_1,44_ = 0.134; *p* = 0.716). A higher average concentration of OBs·mL^−1^ was found in meconia (Table 1).

### 3.2. Infection of L_2_ S. exigua with Suspensions of OBs Obtained from C. carnea Excretion Products

**Figure 1:**
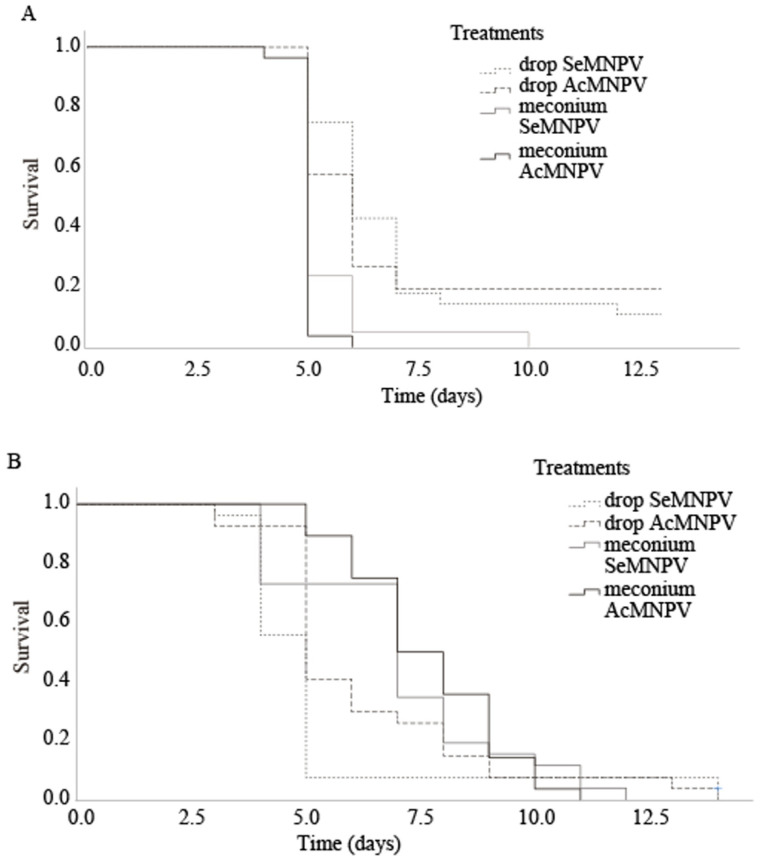
(**A**) and (**B**) Survival curve of *Spodoptera exigua* L_2_ infected with OBs present in predator’s excreta. (**A**) infected with suspensions of OBs; (**B**) infected directly with predator’s excreta.

The OBs recovered from both excretion paths of *C. carnea* showed to be infective for *S. exigua* larvae in all cases (Table 2). Both AcMNPV and SeMNPV meconium treatments caused 100% mortality, as it was expected due to the high OBs concentrations present in the inoculum used. However, drop treatment caused 78.6 ± 9.2% mortality for AcMNPV vs. 85.7 ± 5.8% for SeMNPV. A log-rank test revealed significant differences in speed-of-kill among treatments (χ^2^ = 33.972, df = 3, *p* < 0.001) (Table 2, Figure 1A). The survival curves (Figure 1A) graphically show how the higher viral concentrations of meconia suspensions accelerated the evolution of the disease compared to drop suspensions. Furthermore, the baculovirus species did not affect speed-of-kill (Table 2). The mortality percentage recorded in the negative control (not represented in the figures) was 10.71 ± 3.57 at day 13 (ending point of the larval stage).

### 3.3. Infection of L_2_ S. exigua via Direct Deposition of C. carnea Excretion Products

When the excretion products of *C. carnea* directly contaminated the *S. exigua* artificial diet, no OBs quantification was done due to the characteristics of the procedure. All treatments reached 100% mortality in every repetition, except from SeMNPV drops, which caused 96.4 ± 3.6%. The survival curves (Figure 1B) graphically display the evolution of the disease. Significant differences between treatments were revealed by a log-rank test (χ^2^ = 12.418, gl = 3, *p* = 0.006) (Table 2, Figure 1B). SeMNPV and AcMNPV drops did not differ in speed-of-kill. In contrast, SeMNPV drops treatment was faster than meconium treatments. There are no differences between AcMNPV drops, SeMNPV meconium, and AcMNPV meconium (Table 2). Control mortality was 7.14 ± 4.12% at day 13 (ending point of larval stage).

### 3.4. Chrysoperla carnea Fitness Evaluation after Prey-Mediated Ingestion of SeMNPV and AcMNPV

Regarding the parameters assessed during the larval period (Table 3), the consumption rate did not differ in a statistically significant manner amongst treatments, obtaining average results between 8 and 9 L_2_-*S. exigua* larvae consumed per day by each single *C. carnea* larva. Despite this fact, a significant delay was observed in the developmental time of the L_3_ instar of *C. carnea* between baculovirus treatments and, in comparison with the healthy larvae treatment (control), they needed a half day and a day more to pupate compared to an uninfected-larvae feeding regime. Predator survival until pupation was similar in all feeding regimes with percentages over 70% in all cases (Table 3). In the pupal stage, only AcMNPV treatment extended the duration of this period with statistically significant differences compared to SeMNPV and healthy larvae treatments. Moreover, both BVs treatments reduced the weight of pupae compared to control.

When the imaginal parameters were measured (Table 4), only the preoviposition period showed statistically significant differences. The beginning of oviposition lasted more than one day in the AcMNPV-infected larvae treatment in comparison with SeMNPV-infected and healthy prey. In contrast, none of the rest of the parameters assessed (emergence, sex ratio, fecundity, fertility, adult weight) presented statistically significant differences between treatments.

### 3.5. Chrysoperla carnea Choice Tests

*Chrysoperla carnea* larvae showed no preferences between healthy *S. exigua* larvae and larvae infected by either of the two baculoviruses tested in this study (SeMNPV and AcMNPV) (Table 5). In contrast, the predator showed a significant preference for *S. exigua* larvae compared to *M. euphorbiae* in all cases, regardless of whether these larvae were infected or they were healthy. However, in no case were there significant differences in the searching time spent by the predator for prey catch.

## 4. Discussion

We have reported that predator L_3_ larvae feed on infected *S. exigua* larvae in the presence of healthy prey (*S. exigua* larvae or *M. euphorbiae* adult), while the indirect ingestion of the BVs does not produce significant negative effects on its life cycle. In the meantime, *C. carnea* larvae excrete viable OBs through continuous drop production and when they expel the meconium in the adult molt. Similar findings that BVs may be transmitted through meconia produced by lacewing larvae after consuming infected lepidoptera larvae have been reported for the combination of *C. carnea* and *Heliothis virescens* Fabricius (Lepidoptera: Noctuidae) [14], but they were not found for the combination of *C. rufilabris* (Burmeister) and *Spodoptera frugiperda* Smith [15]. In the latter case, it was suggested that the BV is inactivated in the gut of *Chrysoperla* larvae. So far, the virulence of *C. carnea* larval excreta after consuming SlMNPV and AgMNPV-infected larvae was tentatively assessed by [16], who observed up to 70% mortality of *Spodoptera littoralis* Boisduval and *Anticarsia gemmatalis* Hübner.

However, none of these previous studies have simultaneously compared the two different excretion paths. A direct application of excretion products has not been evaluated either so far.

After five days of feeding on infected prey, we reported a greater viral load of meconia compared to larval excretion drops. This result was foreseeable, since the droplets are continuously produced along the larval stage, while the meconium is solid debris, which concentrates the insoluble remnants until its ejection after adult emergence [9,10]. The higher OBs concentration resulted in a significantly higher speed-of-kill of meconial treatment when we infected *S. exigua* larvae but only when both excreta products are provided suspended in distilled water. Conversely, the direct deposition of the larval drops (after two days of feeding on infected prey) compared to meconium showed a quicker mean lethal time, which can be related to its different matter state, liquid and solid, respectively. The liquid nature of drops could promote a faster ingestion by *S. exigua* larvae. These differences could still be higher in a real scenario, because the accidental ingestion of meconia is more unlikely because of its lower production.

*Chrysoperla carnea* third instar larvae did not discriminate between healthy and baculovirus-infected *S. exigua* larvae, regardless of the BV tested. These results are in agreement with [14] and [15]. In addition, although lacewings are mainly considered homopterophilic [10], when an alternative prey such as *M. euphorbiae* apterous adults was offered vs. both healthy or virus-infected *S. exigua* larvae, the balance was tipped in favor of the latter. Ref. [11] also reported that the highly voracious lacewing third instar had a pronounced preference for healthy larvae of *Pieris brassicae* Linnaeus (Lepidoptera: Pieridae) over aphids of *Brevicoryne brassicae* Linnaeus (Hemiptera: Aphididae). In fact, in lacewing larvae, the discovery of possible prey occurs randomly, and it only appears to be stimulated by several factors within a very short distance [10]. In the current study, this issue is reflected in the searching time necessary for the first attack; in any case, significant differences were observed, which indicates the unpredictable predatory behavior of *C. carnea* larvae. After the first contact, predation success depends upon the resistance of the prey. In that sense, the results obtained are a consequence of the similar level of resistance offered by healthy and 3 d.p.i. virus-infected *S. exigua* larvae, which is mainly due to the comparatively smaller size of both in regard to third instar *C. carnea* larvae.

A strict diet of prey infected with SeMNPV and AcMNPV hardly had a negative impact on the predator, being consistent with previous findings which demonstrate that BVs are specific to a few lepidopteran species [15,16,26]. The negative effects observed are probably related to the nutritional quality of NPV-infected *S. exigua* larvae. In other predators, a reduction in fitness has been observed when consuming nutritionally suboptimal prey [27,28,29]. In fact, parasites may affect the predator’s energy gained from consuming infected prey compared to non-infected prey [30], and in consequence, they may alter some biological parameters.

In any case, the adverse effects observed as a consequence of infected *S. exigua* consumption are expected to be reduced in a field scenario due to the widest prey spectrum, which is not exclusively attached to BV-infected individuals as under laboratory conditions.

## 5. Conclusions

The results of this work show the potential for *C. carnea* to spread BVs at the same time that it preys on pest populations, proving that this predator can be compatible and complementary with the use of BV-based insecticides in Integrated Pest Management field programs. In the case of coincidence of the virus and the predator in the crop, the occasional predation on infected larvae is expected, therefore contributing to the horizontal transmission of the virus.

Thus, an integrated use of both control strategies (macro and microbiological control) could be achieved by the releasing of predator’s larvae previously fed with a BV-sprayed commercial diet. In this way, these larvae would act as BV vehicles for an early application instead of the flood spraying that is usually done. The mobility and adaptability of *C. carnea* larvae, together with their commercial availability, make this species an excellent candidate to explore its potential for BV application under field conditions.

## Figures and Tables

**Table 1:** Presence of occlusion bodies (OBs) in *Chrysoperla carnea* excretion products.

	Drop	Meconium
SeMNPV	1.06 × 10^6^ ± 1.38 × 10^5^	5.11 × 10^6^ ± 6.06 × 10^5^
AcMNPV	8.58 × 10^5^ ± 5.99 × 10^4^	5.14 × 10^6^ ± 2.62 × 10^5^

**Table 2:** MTD (Median Time to Death) values estimated in *S. exigua* second instars infected with suspensions of OBs or directly with predator’s excreta.

Treatment	Infected with Suspension of OBs			Infected Directly with OBs		
	MTD ^1^ (Days)	95% Confidence Limits		MTD ^1^ (Days)	95% Confidence Limits	
SeMNPV drops	6 a	5.4	6.6	5 a	4.8	5.2
AcMNPV drops	6 a	5.4	6.6	5 ab	4.3	5.7
SeMNPV meconia	5 b	4.7	5.3	7 b	5.6	8.4
AcMNPV meconia	5 b	4.9	5.1	7 b	6.1	7.9

^1^ MTD: Median Time to Death. Values followed by letters differ significantly (*p* < 0.05).

**Table 3:** Side effects of the baculoviruses of *S. exigua* (SeMNPV) and *Autographa californica* (AcMNPV) on the preimaginal parameters of *Chrysoperla carnea* after the consumption of healthy and LC_95_-infected L_2_ *Spodoptera exigua* larvae.

Treatment	L_3_ Instar		Pupal Stage		
	Consumption Rate ^1^	Developmental Time (Days) ^2^	Pupation *n* (%) ^3^	Developmental Time (Days) ^4^	Pupal Weight (mg) ^5^
HL	8.70 ± 0.10 a	4.59 ± 0.15 a	44 a (78.60)	9.62 ± 0.10 a	10.56 ± 0.21 a
SeMNPV-IL	8.52 ± 0.09 a	5.08 ± 0.16 b	50 a (89.30)	9.70 ± 0.11 a	9.65 ± 0.22 b
AcMNPV-IL	8.69 ± 0.10 a	5.96 ± 0.17 c	50 a (89.30)	11.09 ± 0.11 b	9.45 ± 0.24 b

*p* < 0.05 indicates significant differences among treatments. Means within columns followed by different letter are significantly different (LSD test). HL = Healthy Larvae. IL = Infected Larvae. ^1^ Larvae consumed daily (total number of larvae daily offered = 10), F_2,141_ = 1.13, *p*= 0.327; ^2^ Duration of the third larval stage, F_2,141_= 18.30, *p* < 0.001; ^3^ Number and percentage of formed pupae, *X*^2^_2_ = 3.50, *p* = 0.174; ^4^ Duration of the pupal stage, F_2,131_ = 61.91, *p* < 0.001; ^5^ F_2,131_ = 7.49; *p* < 0.001.

**Table 4:** Side effects of the baculoviruses SeMNPV and AcMNPV on the imaginal parameters of *Chrysoperla carnea* after consumption of healthy and LC_95_-infected L_2_ *Spodoptera exigua* larvae.

Treatment	Emergence ^1^ *n* (%)	Females/Males (*n*) ^2^	Preoviposition (Days) ^3^	Fecundity (Eggs/Day^−1^) ^4^	Fertility (%) ^5^	Adult Weight (mg)	
						♀ ^6^	♂ ^7^
HL	42 a (95.50)	22/20 a	5.25 ± 0.13 a	18.51 ± 3.43 a	85.56 ± 2.00 a	21.41 ± 0.65 a	8.57 ± 0.25 a
SeMNPV-IL	46 a (92.00)	16/30 a	5.50 ± 0.15 a	10.75 ± 1.44 a	82.52 ± 6.77 a	21.88 ± 0.69 a	8.50 ± 0.28 a
AcMNPV-IL	46 a (92.00)	17/29 a	6.67 ± 0.31 b	14.36 ± 3.20 a	83.26 ± 3.25 a	22.69 ± 0.63 a	8.33 ± 0.27 a

*p* < 0.05 indicates significant differences among treatments. Means within columns followed by different letters are significantly different (LSD test). HL = Healthy Larvae. IL = Infected Larvae. ^1^ Number and percentage of emerged adults. *X*^2^_2_ = 0.48; *P* = 0.788; ^2^ *X*^2^_2_ = 3.44; *p* = 0.179; ^3^ F_2,33_ = 12.33; *p* < 0.001; ^4^ F_2,33_ = 1.51; *p* = 0.235; ^5^ F_2,21_ = 0.10; *p* = 0.905; ^6^ F_2,33_= 0.98; *p* = 0.387; ^7^ F = _2,33_ = 0.22; *p* = 0.807.

**Table 5:** Assessment of prey preference habits of *Chrysoperla carnea* L_3_ larvae under different choice scenarios: infected vs. healthy *S. exigua* larvae, infected *S. exigua* larvae vs. *M. euphorbiae*, and healthy *S. exigua* larvae vs. *M. euphorbiae*.

**Infected vs. Healthy *S. exigua* Larvae**				
	**SeMNPV**		**AcMNPV**	
	**First Attack**	**Searching Time**	**First Attack**	**Searching Time**
Infected larvae	20 a	9.96 ± 2.14 a	21 a	6.70 ± 1.93 a
Healthy larvae	22 a	8.75 ± 1.41 a	19 a	8.24 ± 1.62 a
	*X*^2^_1_ = 0.95; *p* = 0.758	F_1,40_ = 0.23; *p* = 0.635	*X*^2^_1_ = 0.10; *p* = 0.752	F_1,37_ = 0.38; *p* = 0.543
**Infected *S. exigua* Larvae vs. *M. euphorbiae***				
	**SeMNPV**		**AcMNPV**	
	**First Attack**	**Searching Time**	**First Attack**	**Searching Time**
Infected larvae	33 a	7.63 ± 1.41 a	28 a	8.55 ± 1.43 a
*M. euphorbiae*	7 b	11.66 ± 2.98 a	12 b	9.86 ± 1.92 a
	*X*^2^_1_ = 16.90; *p* < 0.001	F_1,38_ = 1.44; *p* = 0.238	*X*^2^_1_ = 6.40; *p* = 0.011	F_1,37_ = 0.27; *p* = 0.609
**Healthy *S. exigua* Larvae vs. *M. euphorbiae***				
	**First Attack**		**Searching Time**	
Healthy larvae	34 a		14.01 ± 9.35 a	
*M. euphorbiae*	12 b		11.01 ± 6.86 a	
	*X*^2^_1_ = 5.58; *p* = 0.018		F_1,45_ = 1.02; *p* = 0.317	

Observations were done for up to 30 min. *p* < 0.05 indicates significant differences among treatments. Means within columns followed by different letters are significantly different.
